# Subcapsular Biloma Secondary to Gallbladder Perforation in Acute Cholecystitis: A Case Report

**DOI:** 10.7759/cureus.97177

**Published:** 2025-11-18

**Authors:** Angelica Spence, Emmanuel Itobi, Christian Wakefield

**Affiliations:** 1 General Surgery, Royal Hampshire County Hospital, Winchester, GBR

**Keywords:** acute cholecystitis, biliary complications, gallbladder perforation, hepatic subcapsular biloma, laparoscopic cholecystectomy, percutaneous drainage, rare biliary complications

## Abstract

Hepatic subcapsular biloma is a rare complication of gallbladder perforation in acute cholecystitis. We report the case of an 87-year-old man who presented with right upper quadrant pain, a new oxygen requirement, and deranged liver function tests, with a recent history of conservatively managed acute calculous cholecystitis. Magnetic resonance cholangiopancreatography (MRCP) demonstrated a thick-walled gallbladder containing a 2 cm calculus with focal fundal perforation and a large subcapsular fluid collection extending beneath the hepatic capsule, along with an associated right pleural effusion. These findings raised suspicion for gallbladder perforation with subcapsular biloma. Due to worsening inflammatory markers despite antibiotic therapy, the patient underwent ultrasound-guided transhepatic drainage, which confirmed the diagnosis by aspirating bile. One week later, he underwent laparoscopic cholecystectomy with intraoperative cholangiography, which confirmed the perforation site and demonstrated normal biliary anatomy. The patient recovered well; drains were removed sequentially, and he was discharged home with outpatient follow-up. This case highlights that a staged approach combining percutaneous drainage and laparoscopic cholecystectomy is a safe and effective management strategy for gallbladder perforation with subcapsular biloma.

## Introduction

Acute calculous cholecystitis is a common general surgical presentation, caused by obstruction of the cystic duct by gallstones [1]. While most patients recover well with timely intervention, gallbladder perforation occurs in approximately 2% of cases and represents an uncommon but serious complication associated with significant morbidity [2]. Diagnostic delays are common because its clinical features can closely resemble those of uncomplicated cholecystitis, and early recognition is crucial to prevent bile leakage, abscess formation, and severe biliary sepsis [3].

When perforation occurs, bile may leak from the gallbladder and form a localised collection. One rare manifestation is the formation of a hepatic subcapsular biloma, in which bile accumulates beneath the hepatic capsule, creating a collection between the capsule and the liver parenchyma [4]. Spontaneous subcapsular biloma secondary to gallbladder perforation, in the absence of trauma or prior intervention, is exceptionally rare, with only a handful of cases reported in the literature [5].

We describe such a case in an elderly patient, highlighting prompt diagnosis and successful management through a staged approach involving percutaneous drainage followed by laparoscopic cholecystectomy.

## Case presentation

An 87-year-old man presented with a four-day history of progressively worsening right upper quadrant (RUQ) pain, reduced appetite, one episode of vomiting, and dark urine. The pain was described as sharp, colicky, and similar to an episode of acute cholecystitis two months earlier, when he had been admitted, treated conservatively and listed for elective laparoscopic cholecystectomy. He had a history of hypertension and known gallstones, regularly took amlodipine and omeprazole, and lived independently with his wife.

On examination, he was tachypnoeic (respiratory rate 28) and hypoxic (oxygen saturations of 93% on 2 L). Abdominal examination revealed a positive Murphy’s sign, RUQ guarding, and sharp referred shoulder-tip pain on deep inspiration. Admission blood tests showed white cell count (WCC) 11.5 ×10⁹/L, CRP 74 mg/L, alanine aminotransferase (ALT) 145 U/L, alkaline phosphatase (ALP) 301 U/L, and bilirubin 59 µmol/L. Intravenous co-amoxiclav was started, and MRCP was performed.

**Figure 1:**
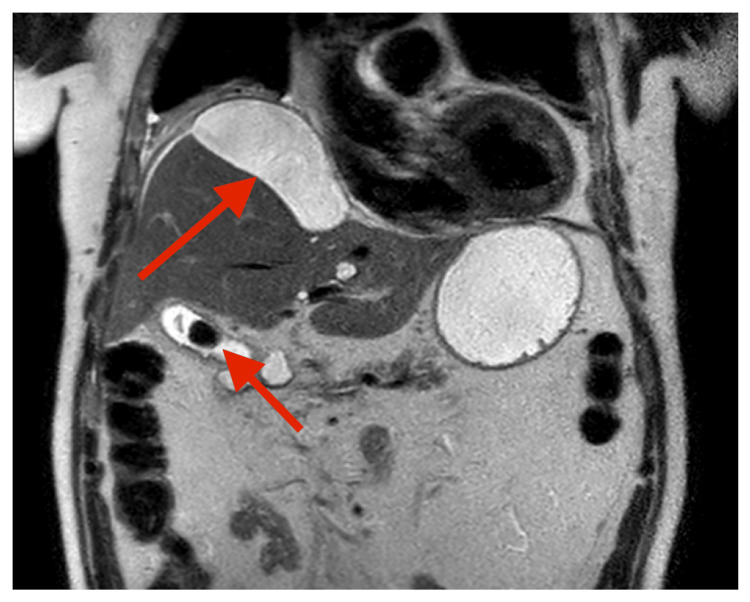
MRCP image showing a thick-walled gallbladder containing a 2 cm calculus, with the extent of the subcapsular biloma. MRCP: Magnetic resonance cholangiopancreatography.

**Figure 2:**
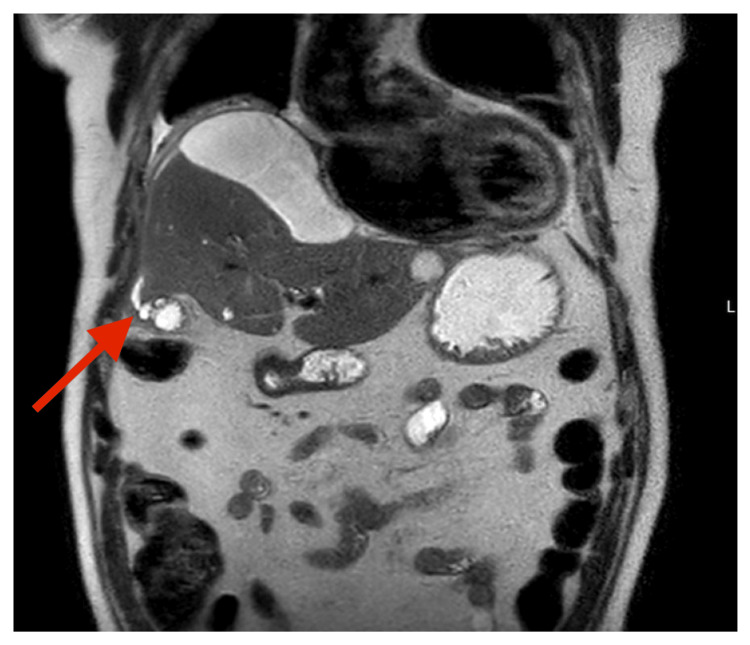
MRCP image demonstrating the focal fundal perforation of the gallbladder, confirming the site of perforation. MRCP: Magnetic resonance cholangiopancreatography.

MRCP demonstrated a thick-walled gallbladder containing multiple calculi, the largest measuring 2 cm (Figure 1). Appearances suggested a focal fundal perforation (Figure 2), with fluid extending beneath the hepatic capsule to form a 10 × 4 cm subcapsular collection. Associated findings included elevation of the right hemidiaphragm, right lower lobe collapse/consolidation, and a right pleural effusion. No intra- or extrahepatic duct dilatation or choledocholithiasis was seen. Multiple simple hepatic cysts were also noted. These findings raised suspicion for gallbladder perforation with subcapsular biloma.

By this point, his inflammatory markers had worsened (CRP 316 mg/L, WCC 18.8 ×10⁹/L), and antibiotics were escalated to intravenous piperacillin-tazobactam. Ultrasound assessment of the biloma initially found no safe drainage route due to diaphragmatic elevation. After discussion with interventional radiology, ultrasound-guided transhepatic drainage was ultimately performed under conscious sedation using the Seldinger technique. An 8Fr locking pigtail catheter was inserted, aspirating bile (culture negative) and confirming the diagnosis of biloma. There were no immediate complications.

Seven days later, laparoscopic cholecystectomy with intraoperative cholangiography (American Society of Anesthesiologists (ASA) grade II) was performed. Intraoperatively, the gallbladder was thick-walled and oedematous, with dense adhesions to the colon and omentum. The previously drained biloma had obliterated the right subphrenic space. The gallbladder fundus showed bile-stained granulation tissue at the site of perforation. Following adhesiolysis and dissection of Calot’s triangle, the cystic duct and artery were isolated, clipped, and divided. Intraoperative cholangiography demonstrated normal biliary anatomy with a slightly dilated common bile duct, no filling defects, and free contrast flow into the duodenum. The gallbladder was dissected from the fossa and retrieved in a specimen bag. A 20F subhepatic drain was placed.

Postoperatively, the patient recovered well. The subhepatic drain was removed prior to discharge the following day, and the radiological drain was removed under fluoroscopic guidance three days later. At follow-up, he remained well.

## Discussion

A biloma is a well-demarcated collection of bile outside the biliary system. The majority are iatrogenic, occurring after procedures such as cholecystectomy, endoscopic retrograde cholangiopancreatography (ERCP), or trauma to the biliary tree [6]. Bilomas secondary to gallbladder perforation are exceptionally uncommon, with only a handful reported in the literature [7]. Our case adds to this limited evidence base and demonstrates a successful management approach.

Early diagnosis of gallbladder perforation can be challenging, as clinical features often mimic uncomplicated cholecystitis, and small perforations may not be apparent on imaging [8]. The presence of a biloma may further complicate interpretation, as its radiological features can resemble cysts, abscesses, or even tumours. Ji G et al. reported a giant subphrenic biloma mistaken for a biliary cystic tumour [8], and Huang HW et al. reported a liver haematoma secondary to gallbladder perforation initially mistaken for a ruptured malignant liver tumour [9]. These examples demonstrate the potential for misdiagnosis.

In our case, MRCP suggested a focal gallbladder perforation extending into the subcapsular space, supported by worsening inflammatory markers. Definitive confirmation was achieved by aspiration of bile during ultrasound-guided percutaneous drainage and intraoperative visualisation of the fundal perforation. This integration of MRCP imaging, USS-guided aspiration, and intraoperative confirmation illustrates a safe and practical pathway for establishing the diagnosis.

There are no standardised guidelines for managing biloma following gallbladder perforation [10]. Small, asymptomatic collections may resolve spontaneously, but larger or infected bilomas require intervention [11]. Image-guided percutaneous drainage of hepatic subcapsular bilomas can be curative, as reported by Neto CB et al. and Kalfadis S et al. [12]. In cases of persistent leakage (and high surgical risk), adjunctive endoscopic approaches such as nasobiliary drainage or biliary stenting have been successfully performed [13-14]. In contrast, Limani N et al. described a large extrahepatic biloma requiring laparotomy, during which 3 L of bile and two gallstones were evacuated, followed by cholecystectomy [7].

Our patient underwent staged management with percutaneous transhepatic drainage followed by laparoscopic cholecystectomy with intraoperative cholangiography. This provided source control along with definitive treatment, preventing recurrence. Compared with previous reports, our case highlights the feasibility of a staged approach in a relatively fit elderly patient.

## Conclusions

Gallbladder perforation with subcapsular biloma is a rare complication of acute cholecystitis and should be considered when imaging demonstrates subcapsular collections. While percutaneous drainage is effective for initial stabilisation and diagnostic confirmation, definitive cholecystectomy should be performed when feasible. This case adds to the limited literature on subcapsular biloma and demonstrates that a staged approach combining percutaneous drainage and laparoscopic cholecystectomy can be a safe and effective management strategy in appropriately selected elderly patients. As this report describes a single case, broader applicability should be considered in context and applied with clinical discretion.

## References

[1] Jones MW, Santos G, Patel PJ, O’Rourke MC (2025). Acute cholecystitis. StatPearls [Internet].

[2] Kristo G (2024). An overview of the management of gallbladder perforations. Glob J Surg Case Rep.

[3] Derici H, Kara C, Bozdag AD, Nazli O, Tansug T, Akca E (2006). Diagnosis and treatment of gallbladder perforation. World J Gastroenterol.

[4] Gónzalez-Muñoz A, Ayala D, Amador V (2025). Giant subcapsular hepatic biloma from gallbladder perforation due to blunt abdominal trauma: an inn-usual case report. J Clin Images Med Case Rep.

[5] Rizvi BS, Rajkumar A (2015). Spontaneous biloma: a rare complication of acute cholecystitis. Am J Gastroenterol.

[6] Balfour J, Ewing A (2023). Hepatic Biloma.

[7] Limani N, Misimi S, Nikolovski A (2023). Large biloma as the initial presentation of gallbladder perforation: a case report and literature review. J Surg Case Rep.

[8] Ji G, Zhu F, Wang K, Jiao C, Shao Z, Li X (2017). A giant and insidious subphrenic biloma formation due to gallbladder perforation mimicking biliary cystic tumor: a case report. Mol Clin Oncol.

[9] Huang HW, Wang H, Leng C, Mei B (2024). Formation and rupture of liver hematomas caused by intrahepatic gallbladder perforation: a case report and review of literature. World J Gastrointest Surg.

[10] Quiroga-Garza A, Alvarez-Villalobos NA, Angeles-Mar HJ (2021). Localized gallbladder perforation: a systematic review of treatment and prognosis. HPB (Oxford).

[11] Neto CB, Ferreira AR, Queirós TM (2022). Hepatic subcapsular biloma: a rare complication of laparoscopic cholecystectomy. Indian J Surg.

[12] Kalfadis S, Ioannidis O, Botsios D, Lazaridis C (2011). Subcapsular liver biloma due to gallbladder perforation after acute cholecystitis. J Dig Dis.

[13] Tsai MC, Chen TH, Chang MH, Chen TY, Lin CC (2010). Gallbladder perforation with formation of hepatic subcapsular biloma, treated with endoscopic nasobiliary drainage. Endoscopy.

[14] Mizuno O, Kawamoto H, Fukatsu H (2008). An iatrogenic hepatic subcapsular biloma successfully treated by percutaneous drainage and endoscopic biliary stenting. Endoscopy.

